# Molecular Evidence for the Hybrid Origin of *Ilex dabieshanensis* (Aquifoliaceae)

**DOI:** 10.1371/journal.pone.0147825

**Published:** 2016-01-25

**Authors:** Lin Shi, Naiwei Li, Shuqiong Wang, Yubing Zhou, Weijie Huang, Yuchen Yang, Yongpeng Ma, Renchao Zhou

**Affiliations:** 1 Shunde Polytechnic, Foshan 528333, China; 2 Institute of Botany, Jiangsu Province and Chinese Academy of Sciences, Nanjing 210014, China; 3 State Key Laboratory of Biocontrol and Guangdong Provincial Key Laboratory of Plant Resources, Sun Yat-sen University, Guangzhou 510275, China; 4 Key Laboratory for Plant Diversity and Biogeography of East Asia, Kunming Institute of Botany, Chinese Academy of Sciences, Kunming 650204, China; Wuhan Botanical Garden, Chinese Academy of Sciences, Wuhan, China, CHINA

## Abstract

*Ilex*, the largest genus of dioecious woody plants, is a good study system to assess the role of hybridization in speciation and evolution. *Ilex dabieshanensis*, a tree endemic to Dabieshan Mountains region, was initially described as a new species. Based on morphological intermediacy and sympatric distribution with its putative parental species, *I*. *cornuta* and *I*. *latifolia*, we proposed it as a natural hybrid between them. In this study, we sequenced one chloroplast intergenic spacer (*trnH-psbA*) and two nuclear genes (*gapC* and *nepGS*) in *I*. *dabieshanensis* and its putative parental species to test the hybrid origin hypothesis. Our results showed that there were one to two differentially fixed sequence differences between *I*. *cornuta* and *I*. *latifolia* at the two nuclear genes. Twelve of the 14 individuals of *I*. *dabieshanensis* exhibited additivity in chromatograms on these differentially fixed sites at both nuclear genes, and the remaining two exhibited additivity in chromatograms on the fixed site at only the *nepGS* gene. Except one haplotype of *I*. *cornuta* at the *nepGS* gene, all of the haplotypes of *I*. *cornuta* at the two nuclear genes were well separated from those of *I*. *latifolia*, and most haplotypes of *I*. *dabieshanensis* were shared with those of *I*. *cornuta* and *I*. *latifolia*. Phylogenetic analysis of these haplotypes was largely consistent with haplotype network analysis. *I*. *cornuta* and *I*. *latifolia* differed by two nucleotide substitutions in the chloroplast intergenic spacer, and 12 individuals of *I*. *dabieshanensis* had the same sequences as *I*. *latifolia*, while the remaining two were identical with *I*. *cornuta*. The molecular data provide convincing evidence for the hybrid origin of *I*. *dabieshanensis* and asymmetrical direction of hybridization. One haplotype of *I*. *cornuta* at the *nepGS* gene was nested with those of *I*. *latifolia*, indicating introgression to *I*. *cornuta*.

## Introduction

When closely related species, especially those within a genus, come into contact, hybridization is a common consequence in many plant groups [1,2]. If hybrids have reduced fitness in the habitats of their parental species, they will be restricted to intermediate habitats [3]. The hybrid zones can be maintained by a balance between selection against hybrids and repeated hybridization between parental species. This kind of hybrid zones can not expand if one or both parental species fail to expand its or their ranges. In this situation, the hybrid taxa may be recognized as locally endemic because they span a relatively limited range. Narrow ranges of species can also be caused by recent speciation by lineage splitting on a local scale, or range contraction of previously widespread species. Thus, precise characterization of the number of hybrid taxa within plant groups with a high proportion of narrow ranging species is critical for assessing the role of hybridization in species diversity and evolution.

*Ilex* L. (Hollies) is the sole genus of Aquifoliaceae [4], consisting of 400–600 species in tropical and subtropical to temperate regions of the world [5–8]. It is also the largest genus of dioecious woody plants [6,9]. The main areas of extant diversification of *Ilex* are East Asia and South America [10–12]. Seeds of *Ilex* species are extremely important food for numerous species of birds, and some wild animals [13,14], and thus *Ilex* species play important roles in the local ecosystems. There are many naturally occurring or cultivated interspecific hybrids in this genus [6, 15–19]. Interspecific hybridization and introgression have probably played important roles in the evolution and speciation of *Ilex*, as also suggested by substantial incongruence between the nuclear phylogeny and the plastid phylogeny of this genus [10–12,20].

Many species in this genus have very limited geographic distributions and to date 97 species of this genus have been listed in the ICUN red list of endangered species [21]. In China, there are about 200 species of *Ilex*, which are mostly distributed in Southwest and South China, with the southern slopes of Qingling Mountains being the northern range margin of this genus [7,8]. Among the 200 species, about 150 species are endemic to China. Many species endemic to China occur in very narrow ranges, and one such is *I*. *dabieshanensis* K. Yao et M. P. Deng [22]. *I*. *dabieshanensis* was initially described as a new species and is distributed only in the Dabieshan Mountains region [8,22], a major mountain range located in the boundary between Hubei, Henan and Anhui provinces in Central China. It is usually found in slopes, roadsides and streamsides with an elevation of 100–500 m [8].

In addition to *I*. *dabieshanensis*, there are three other species of *Ilex* in the Dabieshan Mountains region, namely, *I*. *latifolia* Thunb., *I*. *cornuta* Lindl. et Paxt. and *I*. *chinensis* Sims. *I*. *latifolia* is distributed in Central and South China and Japan, with an elevation of 250–1500 m. *I*. *chinensis* has a very similar geographic distribution with *I*. *latifolia*, but with a broader range in elevation (from sea level to 2000 m). *I*. *cornuta* is native to North, Central and East China and Korea with an elevation of 100–1900 m, and is widely cultivated as an ornamental plant in Europe and North America. While *I*. *chinensis* has papery leaves, crenate leaf margins and purple-red flowers, all other three species have thickly leathery leaves, serrated or spiny leaf margins and yellow-green flowers. Compared with the three other species, *I*. *dabieshanensis* is quite rare. It has many morphological characters intermediate between *I*. *latifolia* and *I*. *cornuta*. For example, *I*. *comuta* has the spiny leaf margins with one to two spines per side and one strong reflexed spine in the apex, and *I*. *latifolia* has serrated leaf margins, with over 20 pairs of teeth, while *I*. *dabieshanensis* has slightly revolute and serrated leaf margins, with four to eight pairs of spines. Based on its rare occurrence in the overlapping areas and morphological intermediacy between *I*. *cornuta* and *I*. *latifolia*, we propose that *I*. *dabieshanensis* might be an interspecific hybrid between them.

Verifying the hybrid origins of taxa in question is valuable for studies on taxonomy, evolution and conservation. In the present study, we determined the sequences of one chloroplast intergenic spacer and two low-copy nuclear genes in the four sympatric *Ilex* species in the Dabieshan Mountains region to test the hypothesis that *I*. *dabieshanensis* is a hybrid of *I*. *cornuta* and *I*. *latifolia*. Because introgression is a common consequence of hybridization and can contribute to transfer of adaptive traits between species [23–25], we also aimed to investigate if there is any evidence for introgression between its parental species.

## Materials and Methods

### Plant materials

**Fig 1:**
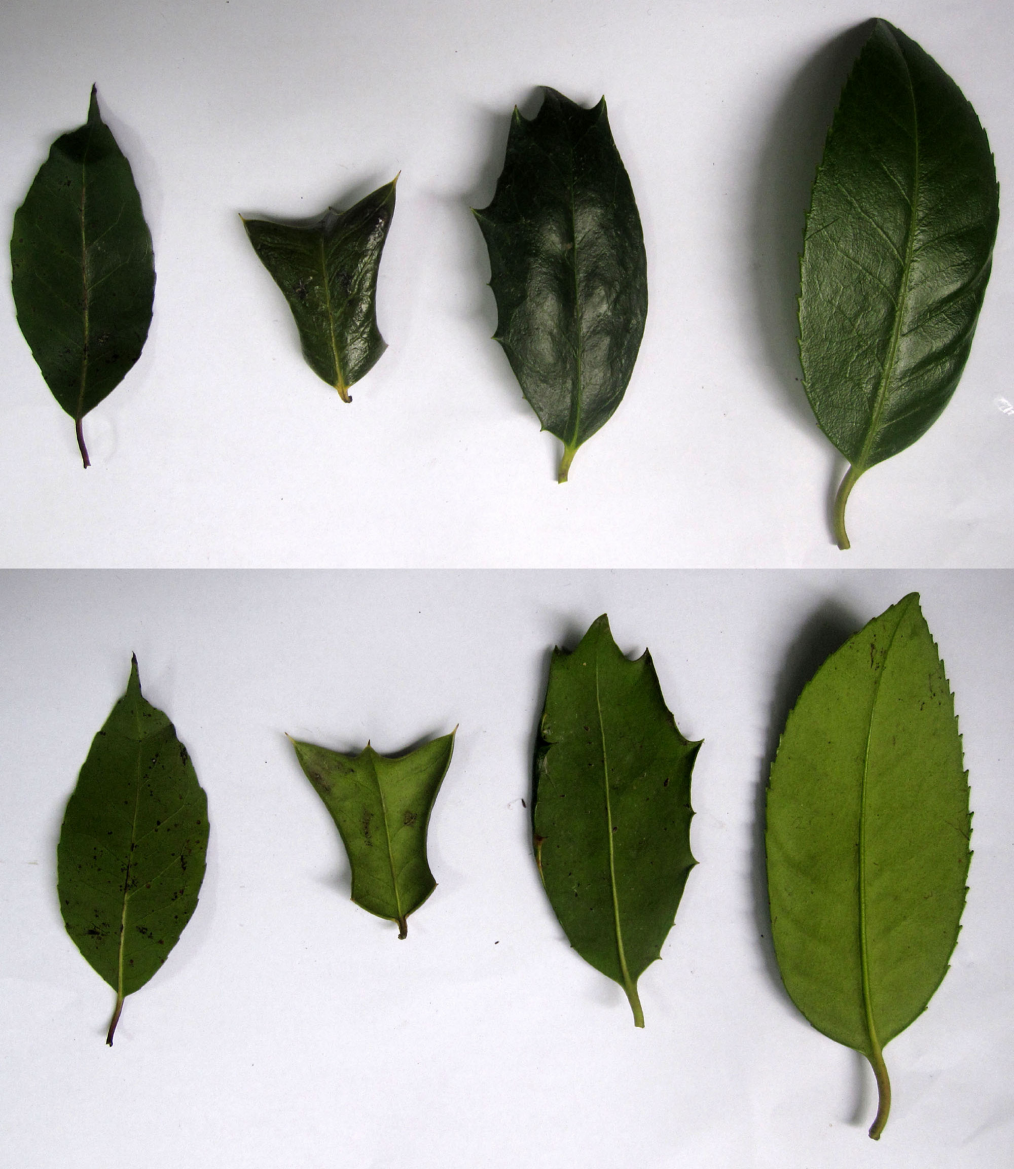
Morphological illustrations for the four *Ilex* species used in this study. The adaxial and abaxial surfaces of the four species are shown in the upper and lower panels, respectively. From left to right are *I*. *chinensis*, *I*. *cornuta*, *I*. *dabieshanensis* and *I*. *latifolia*.

Our sampling site was located in Manshuihe, Huoshan, Anhui, China. At this location, no specific permissions were required for scientific research and no endangered or protected species were involved at this study. These four species were identified according to the diagnostic morphological traits described in the Flora of China [7,8]. Fig 1 showed the morphological illustrations of these species. We sampled 14 individuals of *I*. *cornuta*, 17 indi viduals of *I*. *latifolia* and 14 individuals of *I*. *dabieshanensis*. We used different number of individuals for the three taxa because we could find only these individuals during our field work. We also sampled eight individuals of *I*. *chinensis*, which were used as outgroups. Leaves from each individual of these species were collected and dried in plastic bags with silica gel for subsequent DNA extraction.

### PCR and sequencing of the two nuclear genes and the chloroplast *trn*H-*psb*A regions

We extracted genomic DNA of these samples from dried leaf tissues using the CTAB method [26]. In this study, we used two universal low-copy nuclear genes (*nepGS* and *gapC*), which encode the nuclear encoded plastid glutamine synthetase (nepGS) and the cytosolic glyceraldehyde-3-phosphate dehydrogenase (GAPC), respectively. We obtained the primer sequences of *nepGS* from Emshwiller and Doyle [27]. As for *gapC*, we first used its universal primers of angiosperms [28] for amplification in one individual of each species. After the sequences had been generated, we designed the specific primers for these *Ilex* species. The primer sequences were IgapC-F: 5' TTTGTATTGATATTGCTCCATTTTG 3' and IgapC-R: 5' TGAAATATCCATTCTCACCTGTTG 3'. We amplified the chloroplast *trn*H*-psb*A regions using the universal primers trnH and psbA [29]. We purified the PCR products using a Pearl Gel Extraction Kit (Pearl Bio-tech, Guangzhou, China) and then directly sequenced them on an ABI 3730 DNA automated sequencer with the BigDye chemistry (Applied Biosystems, Foster City, CA, USA). For most individuals of *I*. *dabieshanensis* and some individuals of *I*. *cornuta* and *I*. *latifolia*, direct sequencing produced the superimposed chromatograms on multiple sites and unreadable peaks were observed after intra-individual length polymorphisms at the two nuclear genes, so we used cloning sequencing to phase the haplotypes. We conducted ligation reactions with a pMD18-T&A cloning kit (Takara, Dalian, China) and selected eight positive colonies for each individual for sequencing. We deposited all of the sequences in GenBank with accession numbers KP867812-KP867869 and KP903384-KP903390.

### Sequence analyses

We edited all of the sequences from the four species by using SeqMan (DNASTAR Inc., Madison, WI, USA) and aligned them in Clustal X [30]. At the two nuclear genes, we phased the haplotypes for those individuals that were subject to direct sequencing using Phase [31]. We used Network 4.6 (www.fluxus-engineering.com) with the median-joining method [32] to resolve the relationships of the haplotypes of the nuclear genes. To examine if there is any recombination at the two nuclear genes between the two putative parental species, we detected the minimum number of recombination events for each of *I*. *cornuta* and *I*. *latifolia*, and for the three taxa together, respectively, using DNASP5.0 [33].

For each nuclear gene, we also reconstructed the phylogeny of these haplotypes using maximum parsimony (MP) and maximum likelihood (ML) methods, as implemented in PAUP4.0b [34]. We carried out parsimony analyses by using a heuristic search with tree bisection-reconnection branch swapping, the MulTrees option, accelerated transformation optimization, and 100 random addition replicates. We considered indels as the fifth state, and each indel with two or more nucleotides as a single mutational event. Bootstrap analyses were carried out with 1000 replicates of the heuristic search with simple taxon addition and maxtrees being set to 500. Because *I*. *chinensis* was highly divergent with the three other species at the two nuclear genes and the chloroplast intergenic spacer, we used it as an outgroup to root the phylogenetic trees. For ML analysis, we selected an appropriate nucleotide substitution model for each gene based on the result of Modeltest 3.7 [35]. Similar to the MP analysis, the ML analysis was carried out by using heuristic search with tree bisection-reconnection branch swapping, holding one tree at each step. The maxtrees was also set to 500. Node support was estimated with 1000 bootstrap replicates.

## Results

### Sequence analyses of the chloroplast *trn*H*-psb*A regions in the four species of *Ilex*

The aligned sequences of the chloroplast *trn*H*-psb*A regions from the four species of *Ilex* were 495 bp in length. No within-species variation was found in this region for each of *I*. *cornuta*, *I*. *latifolia* and *I*. *chinenesis*. *I*. *chinensis* differed from *I*. *cornuta* and *I*. *latifolia* by seven nucleotide substitutions and three insertion/deletions (57bp, 2bp and 1 bp, respectively) (Table 1). There were two nucleotide substitutions in the chloroplast *trn*H*-psb*A regions between *I*. *cornuta* and *I*. *latifolia* (Table 1). Of the 14 individuals of *I*. *dabieshanensis* that we sampled, 12 had identical *trn*H*-psb*A sequence with *I*. *latifolia*, whereas the remaining two had the same *trn*H*-psb*A sequences as *I*. *cornuta* (Table 1 and Table 2).

**Table 1:** Variable sites of chloroplast *trnH-psbA* in the four species of *Ilex*. Numbers represent the positions of variable sites. Two haplotypes (H1 and H2) are found in *I*. *dabieshanenesis* and 12 and 2 individuals have H1 and H2, respectively. a: 57 bp deletion; b: 57 bp insertion.

Taxon		Variable sites									
		68	69	83	118	119	127	133–189	295–296	297	302
*I*. *cornuta*		A	A	A	C	A	C	a	GA	A	T
*I*. *latifolia*		A	A	A	A	A	C	a	GA	A	G
*I*. *dabieshanensis*	H1 (12)	A	A	A	A	A	C	a	GA	A	G
	H2 (2)	A	A	A	C	A	C	a	GA	A	T
*I*. *chinensis*		T	C	C	A	C	A	b	- -	T	-

**Table 2:** Haplotypes and genotypes of the 14 individuals of *Ilex dabieshanenesis* at two nuclear genes (*gapC* and *nepGS*) and one chloroplast intergenic region (*trnH-psbA*). Haplotypes with single and double underline have identical sequences with those of *I*. *cornuta* and *I*. *latifolia*, respectively (see Fig 2 for haplotype sharing information). At the chloroplast intergenic region (*trnH-psbA*), H1 and H2 of *I*. *dabieshanenesis* have identical sequences with that of *I*. *latifolia* and *I*. *cornuta*, respectively.

Sample No.	Marker		
	*gapC*	*nepGS*	*trnH-psbA*
Id1	D1, D9	D9, D11	H1
Id2	D1, D10	D5, D8	H1
Id3	D2, D11	D7, D11	H2
Id4	D3, D6	D6, D11	H2
Id5	D5, D9	D3, D4	H1
Id6	D2, D8	D7, D11	H1
Id7	D4, D9	D1, D12	H1
Id8	D13, D14	D5, D13	H1
Id9	D5, D7	D10, D11	H1
Id10	D5, D7	D10, D11	H1
Id11	D5, D7	D10, D11	H1
Id12	D7, D12	D9, D11	H1
Id13	D1, D4	D2, D13	H1
Id14	D1, D15	D5, D11	H1

### Sequence analyses of the *gapC* genes in the four species of *Ilex*

**Fig 2:**
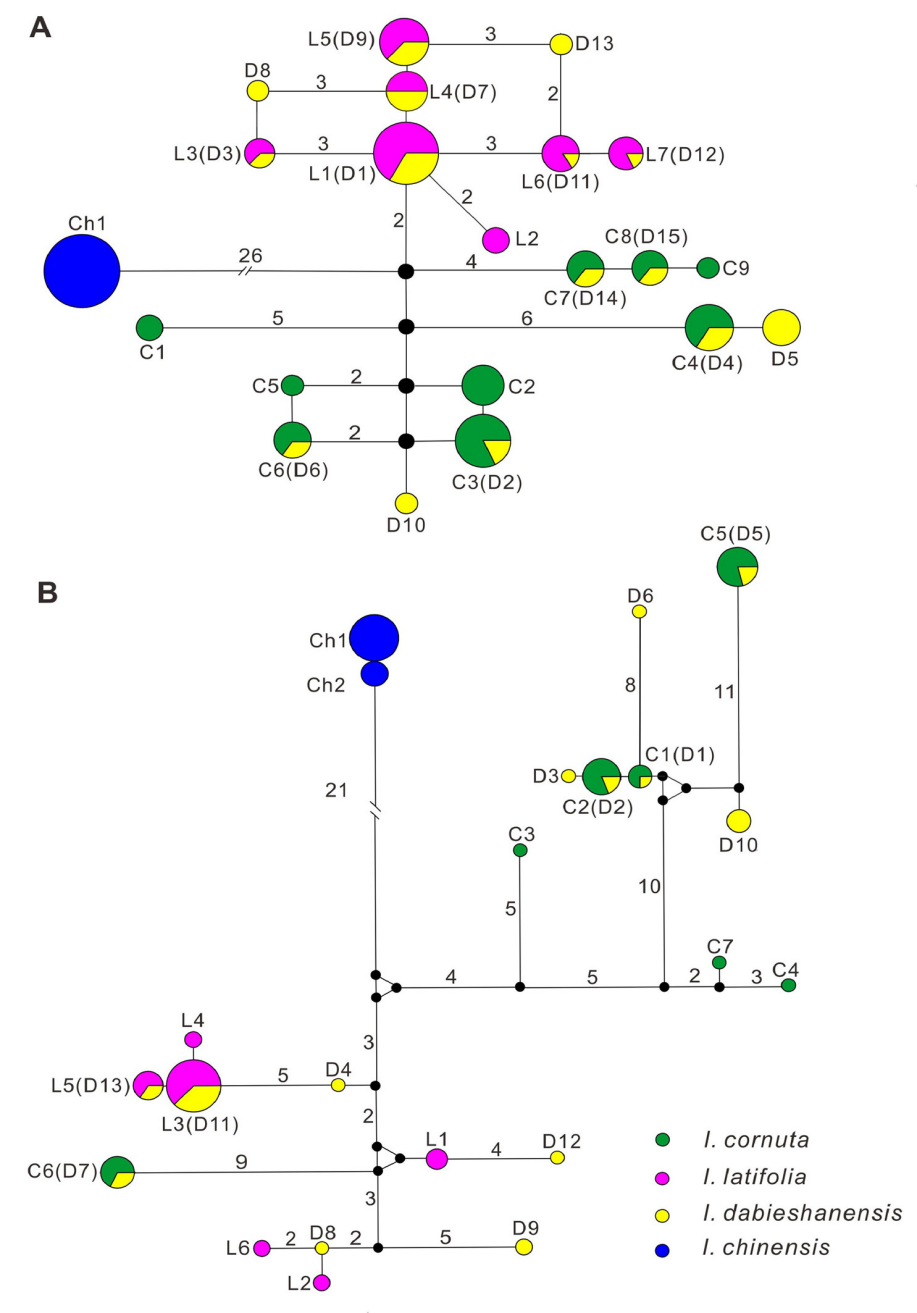
Haplotype networks of *gapC* (A) and *nepGS* (B) genes for four species of Ilex. Green, red, yellow and blue circles represent haplotypes of *I*. *cornuta*, *I*. *latifolia*, *I*. *dabieshanensis* and *I*. *chinensis*, respectively. The circle size denotes the haplotype frequency. C, L, D and Ch denote the haplotype ID for *I*. *cornuta*, *I*. *latifolia*, *I*. *dabieshanensis* and *I*. *chinensis*, respectively. Small black circles represent hypothetical haplotypes. The numbers close to the connecting lines denote mutational steps. The number is not shown when the mutational step is equal to one.

The aligned length of the partial *gapC* gene in the four *Ilex* species was 730 bp. There were 26 fixed nucleotide substitutions between *I*. *chinenesis* and the three other species. Although both *I*. *cornuta* and *I*. *latifolia* had relatively high levels of within-species polymorphism (see below), there were only one fixed nucleotide substitution (the 539^th^ site: T for *I*. *cornuta* and G for *I*. *latifolia*) and one fixed 1-bp insertion/deletion (the 605^th^ site:—for *I*. *cornuta* and T for *I*. *latifolia*) between *I*. *cornuta* and *I*. *latifolia*. For *I*. *dabieshanensis*, 12 of the 14 individuals exhibited chromatogram additivity on these two differentially fixed sites between *I*. *cornuta* and *I*. *latifolia*; the remaining two individuals (Id1 and Id12) had the same sequence as *I*. *latifolia* on these two fixed sites. For the haplotype analysis, no polymorphism at this gene was detected for the eight individuals of *I*. *chinenesis*, while *I*. *cornuta* and *I*. *latifolia* had nine and seven haplotypes, respectively. In contrast, *I*. *dabieshanensis* exhibited much higher haplotype richness, with 15 haplotypes being detected. Eleven of the 15 haplotypes of *I*. *dabieshanensis* were shared with those of *I*. *cornuta* and *I*. *latifolia* (Fig 2A). The remaining four haplotypes were unique to *I*. *dabieshanensis* (Table 2). The haplotypes of *I*. *chinensis* were well separated from those of the three other species (Fig 2A). Recombination analysis showed that the numbers of minimum recombination events in *I*. *cornuta* and *I*. *latifolia* were two and zero, respectively. When the haplotype data of the three taxa (*I*. *cornuta*, *I*. *latifolia* and *I*. *dabieshanensis*) were combined for this analysis, the number of minimum recombination events was three.

**Fig 3:**
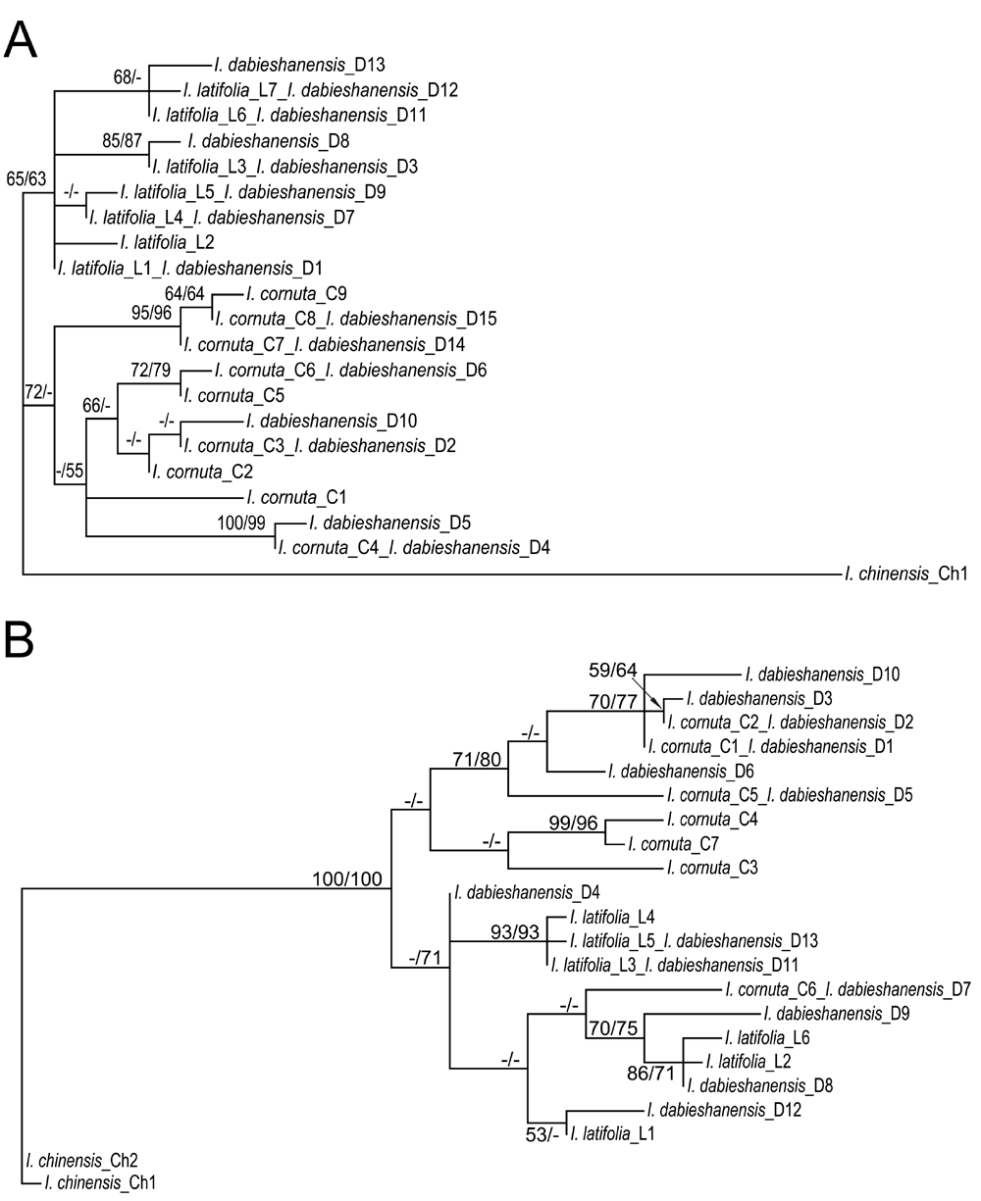
Phylogenetic analyses of haplotype data of two nuclear genes for the four species of Ilex. Shown are the most parsimonious trees for the *gapC* (A) and *nepGS* (B) data sets. Numbers above the branches indicate maximum parsimony/maximum likelihood bootstrap values (> 50%).–is used for < 50% bootstrap support.

Twenty-six of the 730 nucleotide sites were parsimoniously informative for the *gapC* gene matrix. Based on the Akaike Information Criterion in Modeltest, the best-fit model for the ML analysis is the HKY+G model. Phylogenetic analysis with the MP algorithm yielded one most parsimonious tree of 69 steps (Fig 3A; CI = 0.899; RI = 0.889; RC = 0.799). In the MP tree, the haplotypes of *I*. *cornuta*, *I*. *latifolia* and *I*. *dabieshanensis* formed two clades; one clade consisted of all of the haplotypes of *I*. *cornuta* and some halpotypes of *I*. *dabieshanensis*, and the other clade comprised all of the haplotypes of *I*. *latifolia* and other halpotypes of *I*. *dabieshanensis*. The ML analysis of this dataset generated highly similar tree topology with the MP analysis.

### Sequence analyses of the *nepGS* genes in the four species of *Ilex*

The partial *nepGS* gene was 859 bp long in the four species of *Ilex* after sequence alignment. There were 14 fixed nucleotide substitutions between *I*. *chinenesis* and the three other species. Only one differentially fixed nucleotide substitution (the 823^rd^ site: G for *I*. *cornuta* and C for *I*. *latifolia*) were observed between *I*. *cornuta* and *I*. *latifolia*. All 14 individuals of *I*. *dabieshanensis* exhibited chromatogram additivity on this fixed site. For the haplotype analysis, no polymorphism at this gene was detected for the eight individuals of *I*. *chinenesis*. Again, both *I*. *cornuta* and *I*. *latifolia* show relatively high levels of polymorphisms at this gene. Seven and six haplotypes were found in *I*. *cornuta* and *I*. *latifolia*, respectively. Thirteen haplotypes were detected in the 14 individuals of *I*. *dabieshanensis*. Seven of the 13 haplotypes of *I*. *dabieshanensis* were shared with those of *I*. *cornuta* and *I*. *latifolia*, and six haplotypes were unique to *I*. *dabieshanensis* ((Fig 2B, Table 2). Again, the haplotype of *I*. *chinensis* was well separated from those of the three other species at this gene (Fig 2B). The numbers of minimum recombination events at the *nepGS* gene in *I*. *cornuta* and *I*. *latifolia* were four and two, respectively. When the haplotype data of the three taxa (*I*. *cornuta*, *I*. *latifolia* and *I*. *dabieshanensis*) were combined for recombination analysis, the number of minimum recombination events was nine.

Fifty-five of the 859 nucleotide sites were parsimoniously informative for the *nepGS* gene set. Based on the Akaike Information Criterion in Modeltest, the best-fit model for the ML analysis is the TVM+I+G model. Phylogenetic analysis with the MP algorithm yielded one most parsimonious tree of 116 steps (Fig 3B; CI = 0.690; RI = 0.817; RC = 0.564). In the MP tree, the haplotypes of *I*. *cornuta*, *I*. *latifolia* and *I*. *dabieshanensis* formed two well separated clades; one clade consisted of six of the seven haplotypes of *I*. *cornuta* and some halpotypes of *I*. *dabieshanensis*, while the other clade comprised all of the haplotypes of *I*. *latifolia*, one haplotype of *I*. *cornuta* and other halpotypes of *I*. *dabieshanensis*. Again, the ML analysis of this dataset generated highly similar tree topology with the MP analysis.

## Discussion

### The hybrid origin of *I*. *dabieshanensis*

*Ilex dabieshanensis* was initially described as a distinct species in this genus. Since it shows morphological intermediacy between *I*. *cornuta* and *I*. *latifolia*, and occurs where the two species overlap in geographic distribution, we proposed it might be a hybrid between them. In the present study, we sequenced a chloroplast intergenic spacer and two low-copy nuclear genes to test the hybrid origin hypothesis. *I*. *cornuta* and *I*. *latifolia* had one to two differentially fixed sequence differences at both the nuclear and the chloroplast loci. All 14 individuals of *I*. *dabieshanensis* showed an identical chlorotype with either *I*. *cornuta* or *I*. *latifolia* and chromatogram additivity on these differentially fixed sites at one or both nuclear genes, supporting the hypothesis of hybrid origin of *I*. *dabieshanensis*. Two individuals of *I*. *dabieshanensis* (Id1 and Id12) that exhibited chromatogram additivity between *I*. *cornuta* and *I*. *latifolia* at the *nepGS* gene but identical haplotypes with *I*. *latifolia* at the *gapC* gene, must be later-generation hybrids. Furthermore, because chloroplast DNA is maternally inherited in the majority of angiosperms [36] and if it holds in *Ilex*, that 12 and 2 individuals of *I*. *dabieshanensis* have identical *trn*H*-psb*A sequences with *I*. *latifolia* and *I*. *cornuta* respectively indicate that the hybridization was bidirectional and asymmetrical.

We also observed several haplotypes unique to *I*. *dabieshanensis* at the nuclear genes, most of which are only one or two mutational steps from the haplotypes of *I*. *cornuta* or *I*. *latifolia*. This pattern may stem from unsampled polymorphisms in the parental species, which is not surprising given that both *I*. *cornuta* and *I*. *latifolia* have a relatively high level of polymorphism. Of course, we could not exclude the possibility of new mutations occurred in *I*. *dabieshanensis* and intragenic recombination. At each of the two nuclear genes, the number of minimum recombination events in the combined data of the three taxa is higher than the sum of minimum recombination events in *I*. *cornuta* and *I*. *latifolia*, suggesting that intragenic recombination should contribute to this. Together, the scarcity of taxon-specific haplotypes and the evidence of its hybrid origin indicate that *I*. *dabieshanensis* is not a biological species, and therefore, does not merit recognition of the species taxonomic rank.

Interspecific hybridization between *I*. *cornuta* and *I*. *latifolia* may be attributable to several factors. First, *I*. *cornuta* and *I*. *latifolia* have overlapping geographic distributions in Dabieshan Mountains region of Central China. Both species can occur in sparse forests and streamsides, although *I*. *latifolia* usually has higher elevations than *I*. *cornuta* in Dabieshan Mountains region. Second, flowering periods of both species are from April to May [7,8]. Moreover, pollinator sharing between many species of *Ilex* [37] is another factor. Species of this genus are generally dioecious, with male and female flowers on different plants [4,6–8]. Bees and other insects are main pollinators of *Ilex* species [37]. All these factors should contribute to natural hybridization between the two species.

### Potential introgression between *I*. *cornuta* and *I*. *latifolia*

One of the prominent outcomes of natural hybridization is introgression. Introgression can facilitate adaptation by transfer of beneficial genes and corresponding traits [24–26]. Unlike many other interspecific hybrids that often have much reduced fertility, *I*. *dabieshanensis* can produce a great number of seeds and these seeds can germinate [38], thus offering chances for introgression. In this study, at least two individuals of *I*. *dabieshanensis* are identified as later generation hybrids in the hybrid zone, indicative of potential introgression. This is further supported by the *nepGS* gene analysis. Both haplotype network analysis and phylogenetic analysis of the *nepGS* gene reveal that one relatively common haplotype, C6 (D7), of *I*. *cornuta* is nested within the clade of *I*. *latifolia* rather than in its own clade. Introgression of *I*. *latifolia* alleles to *I*. *cornuta* can account for this. Moreover, this haplotype possesses the diagnostic nucleotide G (the 823^rd^ site) of *I*. *cornuta*, which can be generated by intragenic recombination between alleles of *I*. *cornuta* and *I*. *latifolia*, and is also consistent with the occurrence of introgression. Introgression between species of *Ilex* has also been found in previous studies [16–18]. Interestingly, in one case where *I*. *cornuta* and *I*. *integra* hybridize in Wando, Korea, the introgression is also skewed to *I*. *cornuta* [18]. Introgression of alleles of other species to *I*. *cornuta* may contribute to its adaptation to local environments and thus facilitate its range expansion, a hypothesis in need of further testing.

### Natural hybridization in *Ilex*

Besides the species mentioned here, many other species of *Ilex* overlap to different degrees in geographic ranges and flowering periods [4, 6–8]. Furthermore, sharing of pollinators between many *Ilex* species [37] also provides ample opportunities for natural hybridization. Artificial crosses between many species of *Ilex* have proven successful with many combinations producing viable seeds [6]. Thus, reproductive isolation between some *Ilex* species should be incomplete. We propose that many species of *Ilex* that are morphologically intermediate between, and geographically overlapping with, other species, may in fact be of hybrid origin, a hypothesis in need of further testing.

There are approximately 400–600 species in *Ilex* [4–8] and a large proportion of species in this genus is locally, narrowly ranging species. For example, about 150 of the 200 species in China are endemic species [7,8], and many of them are confined to very limited ranges, such as on a small mountain range or on an island. Although the hybrid origin of only two narrowly ranging species, *I*. *dabieshanensis* in this study and *I*. *x wandoensis* in a previous study [18], have been confirmed by molecular means, we believe that many more of these narrowly ranging species probably result from hybridization. This is also suggested by substantial incongruence between the nuclear phylogeny and the plastid phylogeny of this genus [10–12,20].

The fossil record indicates that the genus *Ilex* was cosmopolitan during the Eocene [9,10,39]. Now this genus is mainly distributed in Asia and Central and South America. As the largest genus of dioecious woody plants, *Ilex* is a good system for studying speciation, extinction and biogeography [10–12,20]. Once hybrid taxa of this genus are systematically identified, we can assess the role of hybridization in the species diversity and evolution, and precisely estimate the rate of speciation via lineage splitting in this genus.

## Conclusions

The molecular data clearly demonstrate that *I*. *dabieshanensis* is of hybrid origin and mostly, *I*. *latifolia* is the maternal parent. One haplotype of *I*. *cornuta* at the *nepGS* gene is nested with those of *I*. *latifolia*, indicating introgression to *I*. *cornuta*. Although some narrowly ranging species in this genus, including *I*. *dabieshanensis* in this study, are products of interspecific hybridization, precisely assessing the role of hybridization in the species diversity and evolution in *Ilex* relies on comprehensive identification of hybrid taxa.
